# Muscle-Enriched MicroRNAs Isolated from Whole Blood Are Regulated by Exercise and Are Potential Biomarkers of Cardiorespiratory Fitness

**DOI:** 10.3389/fgene.2016.00196

**Published:** 2016-11-15

**Authors:** Joshua Denham, Priscilla R. Prestes

**Affiliations:** ^1^School of Science and Technology, University of New England, ArmidaleNSW, Australia; ^2^Faculty of Science and Technology, Federation University Australia, Mount HelenVIC, Australia

**Keywords:** myomiR, VO_2 max_, small RNA, epigenetics, non-coding RNA

## Abstract

MicroRNAs (miRNAs) are small non-coding RNA molecules that regulate gene expression post-transcriptionally. Evidence indicating miRNAs influence exercise-induced health and performance adaptations is mounting. Circulating miRNAs are responsible for intercellular communication and could serve as biomarkers for disease and exercise-related traits. Such biomarkers would contribute to exercise screening, monitoring, and the development of personalized exercise prescription. Accordingly, we investigated the impact of long-term strenuous aerobic exercise training and a single bout of maximal aerobic exercise on five muscle-enriched miRNAs implicated in exercise adaptations (miR-1, miR-133a, miR-181a, miR-486, and miR-494). We also determined linear correlations between miRNAs, resting heart rate, and maximum oxygen uptake (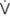
O_2 max_). We used TaqMan assay quantitative polymerase chain reaction to analyze the abundance of miR-1, miR-133a, miR-181a, miR-486, and miR-494 in resting whole blood of 67 endurance athletes and 61 healthy controls. Relative to controls, endurance athletes exhibited increased miR-1, miR-486, and miR-494 content (1.26- to 1.58-fold change, all *p* < 0.05). miR-1, miR-133a, and miR-486 were decreased immediately after maximal aerobic exercise (0.64- to 0.76-fold change, all *p* < 0.01) performed by 19 healthy, young men (20.7 ± 2.4 years). Finally, we observed positive correlations between miRNA abundance and 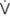
O_2 max_ (miR-1 and miR-486) and an inverse correlation between miR-486 and resting heart rate. Therefore, muscle-enriched miRNAs isolated from whole blood are regulated by acute and long-term aerobic exercise training and could serve as biomarkers of cardiorespiratory fitness.

## Introduction

MicroRNAs (miRNAs) are genetically conserved, small (18–25 nucleotides), non-coding RNA molecules that post-transcriptionally control gene expression by binding to the complementary 3′ untranslated (seed) region of their target messenger RNA (mRNA) molecules, and either promote mRNA degradation or down-regulate translation (Ebert and Sharp, 2012). There are over 2,500 known human mature miRNAs (miRBase release 21, 2014) and each one can have hundreds of mRNA targets (Griffiths-Jones et al., 2008), making miRNAs powerful regulators of gene expression. miRNAs are sensitive to the internal and external environments and are aberrantly expressed in cardio-metabolic disease, including cardiovascular disease (Ikeda et al., 2007; Creemers et al., 2012; Romaine et al., 2015), cancer (Jansson and Lund, 2012), obesity and insulin resistance (Arner and Kulyte, 2015). Circulating miRNAs isolated from the peripheral vasculature are intercellular messengers and could serve as biomarkers of disease (Keller et al., 2011; Creemers et al., 2012; Patnaik et al., 2012; Schultz et al., 2014).

Accumulating evidence indicates acute and long-term exercise training modulates miRNA across multiple organs and plays an important role in health and performance adaptations (Denham et al., 2014; Fernandes et al., 2015). Physiological left ventricular hypertrophy is an adaptation to extensive aerobic exercise training that improves maximum oxygen uptake (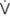
O_2 max_) and a number of miRNAs are implicated in this process (e.g., miR-1, miR-21, and miR-133a; Soci et al., 2011; Ma et al., 2013). Skeletal muscle-enriched miRNAs, called myomiRs, are particularly responsive to aerobic and resistance exercise training (Kirby et al., 2015). For example, 12 weeks of exercise training lead to a decrease in miR-1, miR-133a/b, and miR-206 in conjunction with improvements to 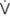
O_2 max_ and insulin sensitivity in healthy young men (Nielsen et al., 2010). miR-494 regulates mitochondrial biogenesis by targeting mtTFA and Foxj3 mRNA and is decreased after short-term exercise in mouse skeletal muscle (Yamamoto et al., 2012).

Circulating miRNAs are also altered by exercise and could serve as biomarkers of exercise-related traits and therapeutic targets to prevent or manage disease (Xu et al., 2015, 2016). For instance, plasma miR-1, miR-133a, miR-206, miR-208, and miR-499 increased immediately after a marathon race and the change in miR-133a abundance was positively associated with 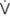
O_2 max_ (Mooren et al., 2014). Similarly, the exercise-induced decrease of serum miR-486 was inversely correlated to 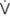
O_2 max_ in 11 young men (Aoi et al., 2013). In a larger study of 100 healthy individuals, those with relatively low 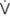
O_2 max_ exhibit increased abundance of serum miR-21, miR-210, and miR-222 (Bye et al., 2013). Therefore, miRNAs and myomiRs, in particular, seem to be influenced by aerobic exercise training, control exercise-induced adaptations, and could be valuable biomarkers of exercise-related traits.

While most of the aforementioned studies have measured circulating miRNAs isolated from serum or plasma (Aoi et al., 2013; Bye et al., 2013; Mooren et al., 2014), others have studied whole blood miRNAs and emphasized their utility as convenient biomarkers for early cancer detection (Bauer et al., 2012; Schultz et al., 2014). The analysis of whole blood miRNAs circumvents some methodological issues when analyzing plasma/serum samples (Schultz et al., 2014). Whole blood provides much higher RNA yields, allows for the analysis of circulating and immune cell miRNAs, and multiple small RNAs can be utilized as experimental endogenous controls. Accordingly, the purpose of this study was to determine whether chronic engagement in strenuous aerobic exercise training and a single bout of maximal aerobic exercise influences the whole blood content of five muscle-enriched miRNAs (miR-1, miR-133a, miR-181a, miR-486, and miR-494), with distinct roles in muscle biology. The concurrent objective was to determine the association between 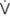
O_2 max_ and miRNAs isolated from whole blood. We hypothesized that acute and chronic exercise training would modulate miRNAs and some would be associated with 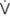
O_2 max_.

## Materials and Methods

### Subjects

A total of 128 subjects were included in the cross-sectional analysis, to determine whether long-term aerobic exercise training was associated with miRNA abundance. The subject characteristics are described elsewhere (Denham et al., 2016). Briefly, all subjects were apparently healthy, non-smokers, free from cardio-metabolic disease and were not taking any medication (**Table 1**). Endurance athletes (*n* = 67) were comprised of endurance runners, cyclists, and triathletes and reported training a minimum of three times a week for 1 year. Healthy controls (*n* = 61) were either inactive or recreational active and did not perform any structured aerobic or resistance exercise training.

**Table 1:** Characteristics of endurance athletes and healthy controls.

Variable	Controls (*n* = 61)	Endurance athletes (*n* = 67)	*p*-value
Men/women (*n*)	47/14	52/15	
Age (year)	28.69 ± 10.64	33.88 ± 10.77	<0.01
Height (cm)	173.82 ± 8.97	176.11 ± 9.69	0.17
Weight (kg)	78.65 ± 10.96	70.61 ± 10.23	<0.001
BMI (weight/height^2^)	26.02 ± 2.95	22.70 ± 2.20	<0.001
Resting HR (beats min^-1^)	68 ± 11	52 ± 8	<0.001
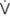 O_2 max_ (ml kg^-1^ min^-1^)	43.73 ± 7.03	58.17 ± 7.85	<0.001


*Data are from two-tailed independent samples *t*-tests and are expressed as mean ± SD.*

*BMI, body mass index; HR, heart rate; 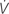
O_*2 max*_, maximum oxygen uptake.*

An additional 19 young men were recruited for the acute exercise trial to determine whether a bout of maximal aerobic exercise influences miRNA abundance. Their characteristics are outlined in **Table 2**. Again, subjects were apparently healthy and not engaged in any structured, intense aerobic or resistance exercise training.

**Table 2:** Characteristics of the men involved in the maximal aerobic exercise trial.

Variable	Subjects
Men (*n*)	19
Age (year)	20.68 ± 2.40
Height (cm)	180.70 ± 6.14
Weight (kg)	80.55 ± 10.66
BMI (weight/height^2^)	24.74 ± 3.75
Resting heart rate (beats min^-1^)	62.55 ± 11.06
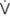 O_2 max_ (ml kg^-1^ min^-1^)	49.53 ± 6.21


*BMI, body mass index; 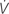
O_*2 max*_, maximum oxygen uptake.*

This study was approved by the Federation University Australia Human Research Ethics Committee and written informed consent was obtained from all subjects.

### Procedures

The study procedures have been outlined previously (Denham et al., 2015, 2016). Briefly, subjects were recruited by word of mouth and flyers posted around the University campus. Subjects included University students and staff, and individuals from the general population, predominantly from the Melbourne, Geelong, and Ballarat regions. Participants were deemed apparently healthy and free from any age-related chronic diseases, according to physical activity readiness questionnaires. All subjects completed a testing session at the Federation University Australia Exercise Physiology Laboratory. Subjects’ height, weight, and body mass index (BMI) were recorded, and a resting blood sample was collected. All subjects then completed a 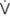
O_2 max_ test as previously described (Denham et al., 2016). The 19 young men involved in the acute exercise trial gave a second blood sample immediately after their 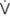
O_2 max_ test.

### MicroRNA quantification

Approximately 20 ml of pre-prandial blood was drawn from the antecubital vein into EDTA tubes using standard phlebotomy procedures. All subjects were instructed to refrain from alcohol or exercise 24–48 h before blood draw. Subjects donated a resting blood sample in the morning (7–10 am) after an overnight fast (8–10 h). RNA was isolated from all samples within 3 h of blood draw to prevent *ex vivo* influences on miRNA abundance. Briefly, 1 ml of blood was washed and centrifuged (2,700 rpm) twice with erythrocyte lysis wash buffer (Qiagen, Australia), and total whole blood RNA was extracted using the miRVana miRNA Isolation Kit following the manufacturer’s guidelines (Thermo Fisher Scientific, Australia). RNA was quantified using the NanoDrop 2000 (Thermo Fisher Scientific, Australia) and reverse transcribed using the TaqMan MicroRNA Reverse Transcription Kit (Thermo Fisher Scientific, Australia) for specific miRNA: hsa-miR-1-3p, hsa-miR-133a-3p, hsa-miR-181a-5p, hsa-miR-486-5p, hsa-miR-494-3p, RNU44, snoU6 (TaqMan IDs: 002222, 002246, 000480, 001278, 002365, 001094, and 001973, respectively, from Thermo Fisher Scientific, Australia). miRNAs were quantified using quantitative polymerase chain reaction using TaqMan assays and thermocycling conditions described previously (Denham et al., 2015). The cycle threshold (Ct) of miRNAs was compared to the geometric mean of endogenous controls, RNU44 and snoU6, and relative abundance was calculated using the 2^-ΔΔCt^ method. The data are graphically represented as either fold difference (FD)/change (FC) or relative abundance.

### Statistical Analyses

All statistical analyses were performed using IBM SPSS Statistics for Mac (Version 23). Two-tailed independent samples *t*-tests were used to determine differences in physical variables and miRNA abundance between athletes and controls, Pearson correlation was used to establish linear relationships between physical traits and miRNA abundance. Two-tailed paired samples *t*-tests were used to identify changes in miRNA abundance in our acute exercise trial cohort. To control for covariates, ANCOVA were used to determine differences in miRNA abundance between athletes and controls. Partial correlations were used to determine linear relationships between physical variables and miRNA, whilst controlling for covariates. Multiple regression analyses were conducted to establish miRNA predictors of exercise-related traits. Statistical significance was set at *p* < 0.05.

## Results

### MicroRNAs and Cardiorespiratory Fitness Parameters

**FIGURE 1:**
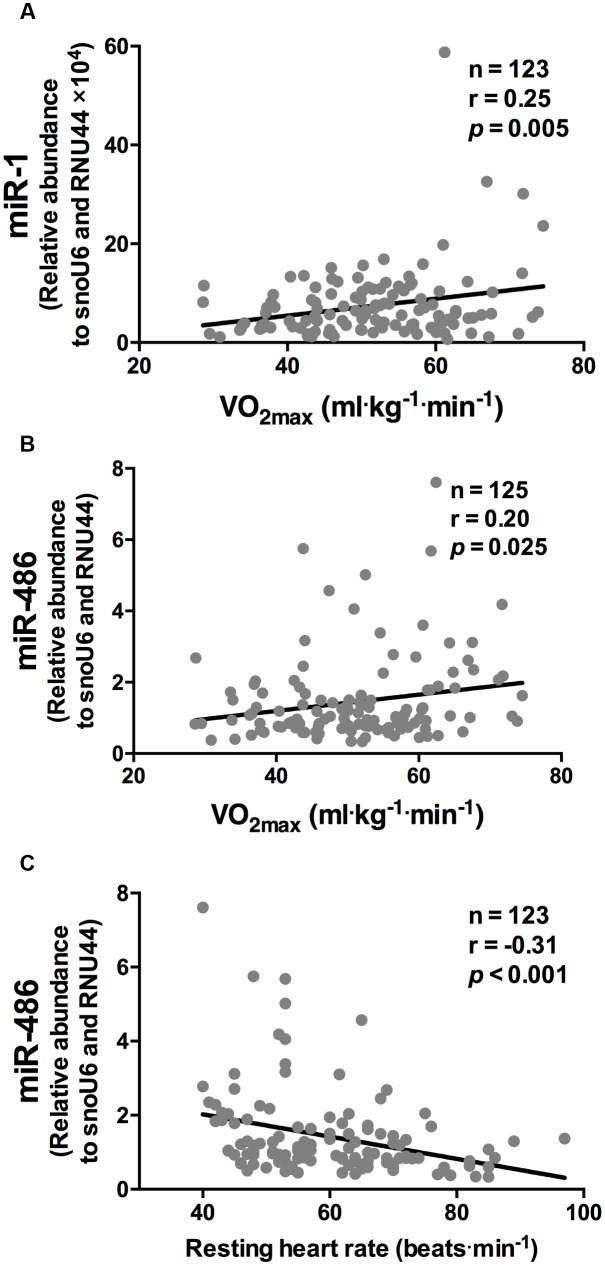
**miR-1 and miR-486 are associated with cardiorespiratory fitness parameters.** While miR-1 **(A)** and miR-486 **(B)** were positively correlated to 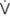
O_2 max_, miR-486 **(C)** was inversely related to resting heart rate (*n* = 123–125). Data are from two-tailed Pearson correlation and expressed as relative abundance normalized to RNU44 and snoU6.

While miR-1 and miR-486 were positively correlated to 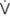
O_2 max_ (*r* = 0.25 and *r* = 0.20, respectively, both *p* < 0.05, **Figures 1A,B**, respectively), miR-486 was inversely correlated to resting heart rate (*r* = -0.31, *p* < 0.001, **Figure 1C**). The positive correlations observed between 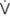
O_2 max_, miR-1, and miR-486 remained statistically significant after adjustment for age, sex, and BMI (*r* = 0.18, *p* = 0.003 and *r* = 0.17, *p* = 0.05, respectively). The inverse correlation between resting heart rate and miR-486 also remained statistical significant after adjustment for age, sex, and BMI (*r* = -0.22, *p* = 0.015). Stepwise regression analyses revealed miR-1 and miR-486, along with age and sex, were independent predictors of 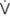
O_2 max_ and explained 24% of the variation (**Table 3**). Furthermore, miR-486, age, and BMI were independent predictors of resting heart rate and also explained 24% of the variation **Table 3**.

**Table 3:** Stepwise regression models for cardiorespiratory fitness parameters.

Dependent variable	Predictors	*B*-value	95% CI	*t*-value	*p*-value	*r*^2^_(adjusted)_
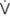 O_2 max_	Age	-0.18	-0.34 to -0.02	-2.26	0.03	0.24
	Sexˆ	-8.66	-12.71 to -4.60	-4.23	<0.001	
	miR-1	0.36	0.12–0.59	3.03	0.003	
	miR-486	1.82	0.41–3.24	2.56	0.01	
Resting heart rate	Age	-0.30	-0.48 to -0.12	-3.21	0.001	0.24
	BMI	1.32	0.69–1.95	4.15	<0.001	
	miR-486	-2.08	-3.74 to -0.43	-2.50	0.01	


*Data are from stepwise linear regression. Variables excluded from the models include: height, miR-133a, miR-181a, and miR-494 (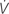
O_*2 max*_); and miR-1, miR-133, miR-181, and miR-494 (resting heart rate).*

*
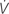
O_*2 max*_, maximum oxygen uptake; BMI, body mass index [weight (kg)/height (cm)^2^]; ˆ female vs. male.*

### MicroRNAs are Differentially Expressed in Endurance Athletes

**FIGURE 2:**
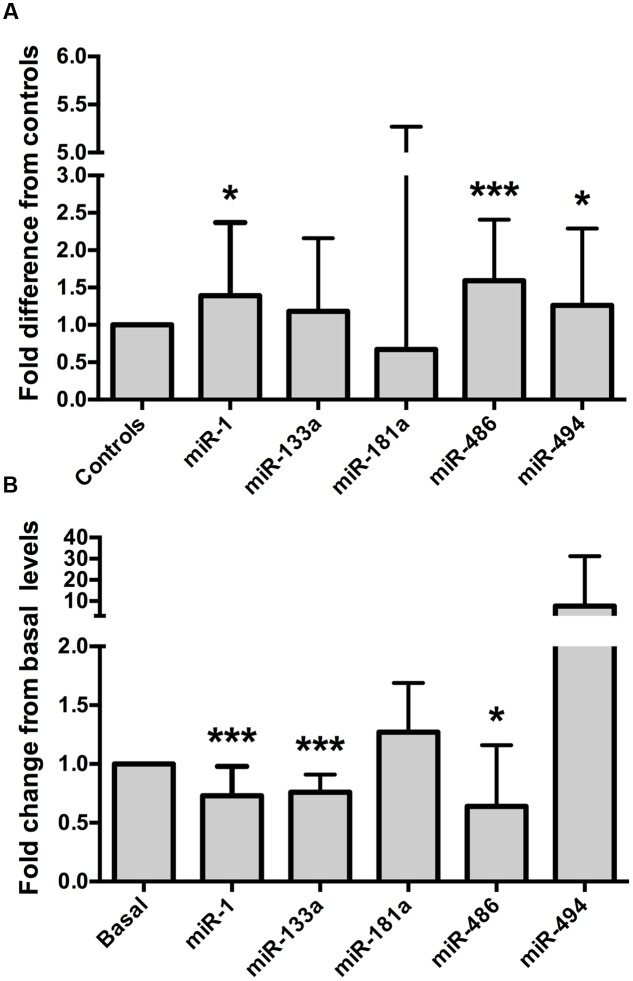
**Muscle-enriched microRNAs are up-regulated in endurance athletes and are controlled by a bout of maximal exercise.** **(A)** Endurance athletes (*n* = 65–67) exhibit increased whole blood miR-1, miR-486, and miR-494 abundance compared to healthy controls (*n* = 56–58, all *p* < 0.05). miR-181a or miR-133a abundance was not statistically different between athletes and controls (both *p* > 0.05, fold difference = 0.67 ± 4.6 and 1.18 ± 0.98, respectively). Data are from two-tailed independent samples *t*-tests using relative abundance and are expressed as fold difference compared to controls. **(B)** Whole blood muscle-enriched miRNAs (miR-1, miR-133a, and miR-486) are down-regulated by a single bout of maximal aerobic exercise. Data are from paired *t*-tests using relative expression and are expressed as fold change relative to basal. The error bars indicate standard deviations. miR, microRNA; 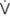
O_2 max_, maximum oxygen uptake. ^∗^*p* < 0.05; ^∗∗∗^*p* < 0.001.

Relative to the healthy controls, endurance athletes were, on average, 5.07 years older, had lower body mass, BMI and resting heart rate, and superior 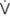
O_2 max_ (all *p* < 0.01, **Table 1**). The intra-assay coefficient of variation (CV) for these miRNA assays ranged from 0.34 to 0.74%. Resting whole blood abundance of miR-1, miR-486, and miR-494 was increased in athletes compared to controls (FD ± SD: 1.38 ± 0.98, 1.58 ± 0.82, and 1.26 ± 1.03, respectively, all *p* < 0.05, **Figure 2A** and **Supplementary Table S1**). There was no statistically significant difference in miR-181a or miR-133a abundance between athletes and controls (both *p* > 0.05, **Figure 2A**). The increased abundance of miR-1, miR-486, and miR-494 in athletes remained statistically significant after adjustment for age and sex (*p* < 0.05, *p* < 0.001, and *p* < 0.05, respectively).

### MicroRNAs are Modulated by One Bout of Maximal Aerobic Exercise

MyomiR abundance was assessed in 19 healthy, young men before and after a 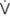
O_2 max_ test, to establish whether miRNAs were regulated by a single bout of maximal aerobic exercise. The characteristics of these men are outlined in **Table 2**. The intra-assay CVs for these miRNA experiments ranged from 0.38 to 0.71%. Maximal aerobic exercise decreased miR-1, miR-133a, and miR-486 abundance (FC ± SD: 0.73 ± 0.24, 0.76 ± 0.15, 0.64 ± 0.52, respectively, all *p* < 0.05, **Figure 2B** and **Supplementary Table S2**). No statistically significant changes were identified for miR-181a or miR-494 after maximal aerobic exercise (FC ± SD: 127 ± 0.42 and 7.63 ± 23.55, respectively, both *p* > 0.05, **Figure 2B**).

## Discussion

This study was designed to establish the influence of acute maximal exercise and long-term involvement in regular strenuous aerobic training on five miRNAs with known roles in skeletal muscle biology. The miRNAs were chosen *a priori* because previous studies indicated they control exercise-induced adaptations to skeletal or cardiac muscle and could serve as biomarkers for exercise-related traits (Nielsen et al., 2010; Yamamoto et al., 2012; Russell et al., 2013; Fernandes et al., 2015; Camera et al., 2016). We are the first to establish that whole blood muscle-enriched miRNAs are predictors of cardiorespiratory fitness parameters and that endurance athletes, who regularly participate in strenuous aerobic exercise, possess elevated levels of whole blood miR-1, miR-486, and miR-494. We also characterized the miRNAs response to a single bout of maximal aerobic exercise and found marked changes in whole blood miRNA (miR-1, miR-133a, and miR-486) abundance. Together, these data indicate muscle-enriched miRNAs isolated from whole blood samples are regulated by a single bout of maximal aerobic exercise and repeated exposure to endurance exercise, implicating them in associated health and performance benefits. These data also suggest whole blood miRNA abundance could be utilized as biomarkers of exercise-related traits (e.g., 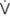
O_2 max_ and resting heart rate).

### Circulating miRNAs Could Control Exercise-Induced Adaptations

Circulating miRNAs could be crucial for the systemic health benefits conferred by exercise as they are responsible for intercellular transcriptional regulation (Chen et al., 2012). Circulating miRNAs include those secreted from blood cells and organs in the body, which are primarily transported in microvesicles or RNA-binding proteins (high-density lipoproteins or argonaute 2; Chen et al., 2012). The miRNAs chosen *a priori* in this study are muscle-enriched miRNAs previously described to regulate important genes in pathways central for skeletal and heart muscle (Wang et al., 2010; Fernandes et al., 2015; Kirby et al., 2015), which made them attractive candidates as exercise biomarkers. For instance, while miR-1 augments myogenesis by deregulating its target mRNA, histone deacetylase (*HDAC4*), miR-133 promotes myoblast proliferation through inhibition of serum response factor (*SRF*) (Chen et al., 2006). Skeletal muscle miR-1 and miR-133a content is markedly reduced during hypertrophy elicited by functional overload (McCarthy and Esser, 2007). miR-1 and miR-486 represses *PAX7* mRNA leading to down-regulation of the genes it controls and, in turn, promotes myoblast proliferation (Chen et al., 2010; Dey et al., 2011). Interestingly, we found increased whole blood content of miR-1, miR-486, and miR-494 in endurance athletes with superior cardiorespiratory fitness levels compared to healthy controls. It is possible that aerobic exercise training up-regulates miR-1 and miR-486 expression to promote myoblast differentiation and proliferation and enable the metabolic reprogramming of skeletal and heart muscle to promote adaptations conducive to physical performance.

Another potential outcome of the increased miR-486 and miR-494 observed in endurance athletes compared to controls is the possibility of physiological cardiac hypertrophy. Physiological cardiac remodeling is an adaptation to extensive aerobic exercise training and facilitates an increased stroke volume, and unlike pathological cardiac hypertrophy, is associated with an improved heart function. Indeed, miR-486 targets phosphatase and tensin homolog (*PTEN*) and forkhead box O1 (*Foxo1a*), which inhibit PI3K/Akt signaling (Small et al., 2010; Xu et al., 2012). *PTEN* is also deregulated by miR-494 (Liu et al., 2010, 2012; Wang et al., 2010). Given the well-established role of PTEN and the PI3K/Akt signaling pathway in physiological left ventricular hypertrophy (Kehat and Molkentin, 2010; Ellison et al., 2012; Ma et al., 2013), increased miR-486 and miR-494 could encourage exercise-induced physiological left ventricular hypertrophy and subsequent augment 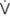
O_2 max_ and endurance performance by deregulating its target, PTEN. This is, however, speculative and should be examined in future work.

Our data contributes to the mounting evidence implicating miRNAs as regulators of exercise-induced adaptations (Zacharewicz et al., 2013; Denham et al., 2014; Kirby et al., 2015). Given miRNAs are responsible for tissue specific gene regulation, it is difficult to compare our data to others because of the novelty of this study examining the impact of long-term endurance exercise on the content of muscle-enriched miRNAs in whole blood. Although whole blood miR-181a abundance was increased after a 30-min exercise session at 80% of peak oxygen uptake (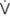
O_2_) in highly trained ski athletes previously (Tonevitsky et al., 2013), the increased miR-181a after maximal exercise in our study did not reach statistical significance. Inverse correlations were observed between miR-181a and the expression of two genes involved in glycogen metabolism, rhophilin associated tail protein 1-like (*ROPN1L*) and solute carrier family 37 member 3 (*SLC37A3*) (Tonevitsky et al., 2013). Likewise, elevated levels of miR-181a in peripheral blood mononuclear cell (Radom-Aizik et al., 2012) and skeletal muscle (Russell et al., 2013) was observed in healthy young men 3 h after following a 30-min cycle interval session and a 60-min cycle at 70% of 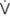
O_2 max_, respectively. Future work will be crucial to elucidate the biological roles whole blood muscle-enriched miRNAs have in response to exercise.

### Whole Blood miRNAs as Potential Biomarkers of Exercise-Related Traits

MicroRNA expression profiles seem to be reflective of exercise status. Our and previously published data (Baggish et al., 2011; Bye et al., 2013; Mooren et al., 2014) support the premise that circulating miRNAs could serve as biomarkers of 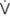
O_2 max_. For example, non-fasted, healthy individuals with relatively low 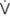
O_2 max_ (*n* = 50) possessed increased plasma miR-21, miR-210, and miR-222 content compared to those with a high 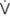
O_2 max_ (*n* = 50) (Bye et al., 2013). Moreover, the response of plasma miR-1 and miR-133a to a marathon event was positively correlated to 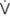
O_2 max_ and speed lactate inflection point in a cohort of 14 male endurance athletes (Mooren et al., 2014). Here, we found novel correlations between whole blood miRNAs, 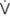
O_2 max_ (miR-1 and miR-486) and resting heart rate (miR-486) in crude and analyses for covariates. To date, this is the largest study to analyze circulating miRNAs in context with 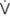
O_2 max_. Thus, whole blood miRNAs are indicative of cardiorespiratory fitness and validation studies will be required to establish their utility as biomarkers.

Others have demonstrated myomiR changes after various forms and intensities of exercise and also after longer periods of exercise training in human plasma (Baggish et al., 2011; Aoi et al., 2013; Nielsen et al., 2014; Wardle et al., 2015; Clauss et al., 2016; Cui et al., 2016) and skeletal muscle (Nielsen et al., 2010; Russell et al., 2013). Many of the findings amongst studies are incomparable due to different subject demographics, tissue sample types, exercise timing and other methodological considerations. Notably, a smaller study investigated whole blood genome-wide miRNA responses to acute exercise in 12 elite endurance athletes and 12 controls and found 24 differentially regulated miRNAs with at least a 1.5-fold change after Benjamini–Hochberg adjustment (Backes et al., 2014). Conversely, we observed dynamic regulation of whole blood miRNAs in response to a single bout of maximal exercise in healthy young men. Unlike the influence that chronic aerobic exercise has on resting miRNA abundance, the response of miRNAs to acute maximal exercise had the opposite effect. Specifically, the increased abundance of miRNAs (miR-1, miR-133a, and miR-486) in endurance athletes compared to the controls was down-regulated after maximal exercise in young men. Our data is consistent with previous findings that acute exercise training decreases serum miR-486 abundance (Aoi et al., 2013). miR-1 and miR-133a seem particularly responsive to aerobic exercise as their content increases in athletes after a marathon race (Clauss et al., 2016), and in young men after high-intensity interval training or distance matched vigorous exercise (Cui et al., 2016). Temporal exercise effects, exercise intensity, and other factors such as methodological differences—sample type and processing, athletic status, etc—are potential explanations for the discrepancy between our findings and that of previous studies (Clauss et al., 2016; Cui et al., 2016). Nonetheless, our data indicates muscle-enriched whole blood miRNA responses are unique to acute and long-term exercise training. Therefore, our preliminary data should encourage the utilization of whole blood miRNA analysis for the identification of biomarkers of exercise-related traits and further studies to elucidate what effects these changes may have on various tissues and exercise adaptations.

### Strengths and Limitations

Our study was the largest to investigate muscle-enriched miRNAs in relation to cardiorespiratory fitness. Moreover, the methodology utilized in the present study allows for the analysis of lowly expressed miRNAs that may not be detected using other genome-wide approaches, such as sequencing and microarrays. Another strength of our study is that we examined the effects of exercise on muscle-enriched miRNAs found in whole blood, which as mentioned previously, overcomes some methodological issues associated with serum and plasma miRNA analyses (Pritchard et al., 2012). There are, however, some limitations that should be noted. Only men were recruited for the maximal aerobic exercise trial. Whilst we found no difference in miRNA abundance between men and women included in the cross-sectional study (data not shown), future work should investigate the immediate effects of a single exercise session on miRNAs in women. The miRNAs analyzed in the present study were chosen *a priori* due to their roles in skeletal muscle and heart biology. A more comprehensive genome-wide analysis of miRNAs regulated by acute and long-term exercise training is also warranted. Future studies are also required to validate the utility of muscle-enriched miRNAs (miR-1, miR-133a, miR-486, and miR-494) and others as biomarkers of exercise-related traits.

## Conclusion

We have established the impact that a single bout of maximal aerobic exercise and long-term exercise training has on whole blood muscle-enriched miRNAs and revealed novel correlations between whole blood miRNAs and cardiorespiratory fitness. These data suggest circulating miRNAs govern physiological adaptations to aerobic exercise training and support the concept of blood-based biomarkers of cardiorespiratory fitness. Future work should identify if other whole blood miRNAs are associated with exercise-related traits, determine their functional roles across multiple tissues and therapeutic utility for disease prevention.

## Author Contributions

JD conceived and designed the study, recruited and tested participants, completed experiments, analyzed and interpreted data, and wrote the manuscript. PP completed experiments, analyzed and interpreted data, and revised the manuscript.

## Conflict of Interest Statement

The authors declare that the research was conducted in the absence of any commercial or financial relationships that could be construed as a potential conflict of interest.

## Abbreviations

BMI: body mass index
CV: coefficient of variation
FC: fold change
FD: fold difference
HR: heart rate
miRNA: microRNA
mRNA: messenger RNA
SD: standard deviation
O_2 max_: maximum oxygen uptake
O_2 peak_: peak oxygen uptake
