# Supplementary material for: Muscle-Enriched MicroRNAs Isolated from Whole Blood Are Regulated by Exercise and Are Potential Biomarkers of Cardiorespiratory Fitness
Source: Front Genet. 2016 Nov 15;7:196. doi: 10.3389/fgene.2016.00196 (PMC5108773; doi:10.3389/fgene.2016.00196)
Supplement: Supplementary file 1 [file Table_1.docx]

| **microRNA** | **Controls** | **Athletes** | ***p*-value** |
| --- | --- | --- | --- |
| microRNA-1 | 5.96 ± 4.22 | 8.66 ± 8.79 | 0.03 |
| microRNA-133a | 14.41 ± 9.77 | 17.13 ± 13.40 | 0.20 |
| microRNA-181a | 87.90 ± 453.26 | 4.87 ± 11.54 | 0.14 |
| microRNA-486 | 1.02 ± 0.51 | 1.83 ± 1.46 | 0.00009 |
| microRNA-494 | 24.83 ± 13.35 | 30.73 ± 14.40 | 0.02 |

Supplementary Table 1. MicroRNA abundance in athletes and controls.

Data are from independent samples t-tests and are expressed as relative abundance (mean ± SD). MicroRNA-1 and microRNA-494 × 10^4^; microRNA-133a and microRNA-181a × 10^3^. MicroRNAs are normalised to the geometric mean of RNU44 and U6 snRNA.
