# Supplementary material for: Muscle-Enriched MicroRNAs Isolated from Whole Blood Are Regulated by Exercise and Are Potential Biomarkers of Cardiorespiratory Fitness
Source: Front Genet. 2016 Nov 15;7:196. doi: 10.3389/fgene.2016.00196 (PMC5108773; doi:10.3389/fgene.2016.00196)
Supplement: Supplementary file 2 [file Table_2.DOCX]

Supplementary Table 2. MicroRNA abundance in young men before and after maximal aerobic exercise.

| **microRNA** | **Before exercise** | **After exercise** | ***p*-value** |
| --- | --- | --- | --- |
| microRNA-1 | 10.55 ± 9.59 | 7.16 ± 6.77 | 0.0009 |
| microRNA-133a | 65.56 ± 46.66 | 51.32 ± 46.20 | 0.0001 |
| microRNA-181a | 105.35 ± 213.36 | 78.97 ± 30.58 | 0.57 |
| microRNA-486 | 4.17 ± 3.59 | 2.32 ± 1.80 | 0.02 |
| microRNA-494 | 355.13 ± 1381.47 | 457.35 ± 1024.59 | 0.70 |

Data are from independent samples t-tests and are expressed as relative abundance (mean ± SD). MicroRNAs are normalised to the geometric mean of RNU44 and U6 snRNA.
